# Progress in Studies on Rutaecarpine. II.—Synthesis and Structure-Biological Activity Relationships

**DOI:** 10.3390/molecules200610800

**Published:** 2015-06-11

**Authors:** Jong-Keun Son, Hyeun Wook Chang, Yurngdong Jahng

**Affiliations:** College of Pharmacy, Yeungnam University, Gyeongsan 712-749, Korea; E-Mails: jkson@ynu.ac.kr (J.-K.S.); hwchang@ynu.ac.kr (H.W.C.)

**Keywords:** alkaloid, rutaecarpine, antiplatelet activity, vasodilatory activity, anticancer activity, anti-cholinesterase activity, anti-obesity activity

## Abstract

Rutaecarpine is a pentacyclic indolopyridoquinazolinone alkaloid found in *Evodia rutaecarpa* and other related herbs. It has a variety of intriguing biological properties, which continue to attract the academic and industrial interest. Studies on rutaecarpine have included isolation from new natural sources, development of new synthetic methods for its total synthesis, the discovery of new biological activities, metabolism, toxicology, and establishment of analytical methods for determining rutaecarpine content. The present review focuses on the synthesis, biological activities, and structure-activity relationships of rutaecarpine derivatives, with respect to their antiplatelet, vasodilatory, cytotoxic, and anticholinesterase activities.

## 1. Introduction

**Figure 1:**
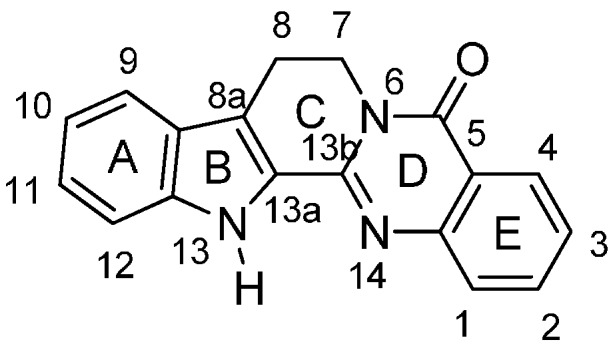
Structure of rutaecarpine (**1a**).

Rutaceous plants, especially *Evodia rutaecarpa* (its dried fruit is called ‘Wu-Chu-Yu’ in China), have long been used to treat gastrointestinal disorders, headache, amenorrhea, and postpartum hemorrhage in traditional oriental medicine [1,2]. The alkaloid, rutaecarpine (8,13-dihydroindolo-[2′,3′:3,4]pyrido[2,1-*b*]quinazolin-5(7*H*)-one, **1a**, Figure 1) was first isolated in 1915 by Asahina and Kashiwaki from an acetone extract of base-treated *Evodia rutaecarpa* [3,4,5] and later from ‘Wu-Chu-Yu’ [6]. Interest in the molecule has since been growing, presumably due to its characteristic structure and intriguing biological properties (733 references were found in the SciFinder database provided by the American Chemical Society). In addition, 55 patents have been issued regarding its isolation, biological activity, synthesis, metabolism, and toxicology. Numbers of papers covering rutaecarpine are summarized in Table 1.

**Table 1:** Numbers of references listed for recent years.

Period	1915–2007	2008	2009	2010	2011	2012	2013	2014	2015	Total
Numbers	339	42	46	43	59	69	64	52	19	733

Of the 17 review papers written to date, eight have focused on the synthesis of rutaecarpine [7,8,9,10,11,12,13,14], seven on pharmacology [15,16,17,18,19,20,21], one on the modulation of cytochrome P450 [22], and one on detection methods [23]. A review published in 1983 by Bergman [7] covers the nomenclature, structure, synthesis and pharmacological properties of rutaecarpine and of related quinazolinone alkaloids. The review of Wang *et al*., written in Chinese in 2006, provides details of the synthesis of rutaecarpine based on construction patterns of the five-ring system [10]. Shakhidoyatov and Elmurado’s review covered the most recent view on the general point of view for tricyclic quinazoline alkaloids [14]. A review written in 1999 by Sheu addressed the *in vitro* and *in vivo* pharmacology of rutaecarpine [15] and later described the cardiovascular pharmacological actions of rutaecarpine in his recent review [20]. More recently, in 2010, Jia and Hu reviewed its cardiovascular protective effects [19]. The present work focuses on the synthesis, biological activities, and structure-activity relationships, with respect to the antiplatelet, vasodilatory, cytotoxic, and anticholinesterase activities, of rutaecarpine derivatives, and complements our first review published in 2008 [24].

## 2. Synthesis of Rutaecarpine

**Scheme 1:**
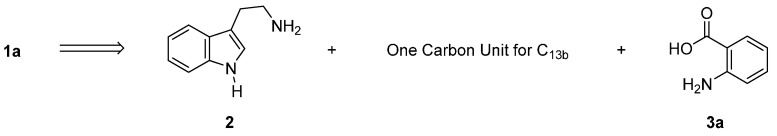
Retrosynthetic analysis for rutaecarpine synthesis.

A simple retrosynthetic analysis leads to tryptamine (**2**) and its equivalents for the indole moiety, and anthranilic acid (**3a**) and its equivalents for the quinazolinone moiety, which leaves an additional one-carbon unit needed for the C_13b_ atom in rutaecarpine (Scheme 1).

**Figure 2:**
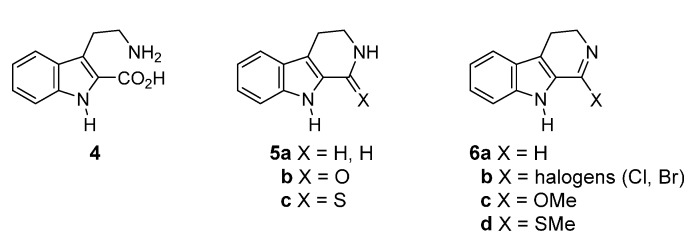
Structures of tryptamine (**2**) equivalents for rutaecarpine synthesis.

Tryptamine (**2**) has been one of most popular starting materials [1,11] and the compounds **4**, **5**, and **6** (Figure 2) have been used as alternative starting materials which provide the A,B,C-ring system and the one-carbon unit at C_13b_.

**Figure 3:**
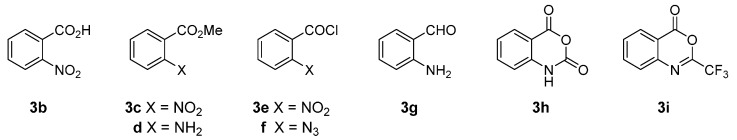
Structures of anthranilic acid (**3**) equivalents for rutaecarpine synthesis.

**Scheme 2:**
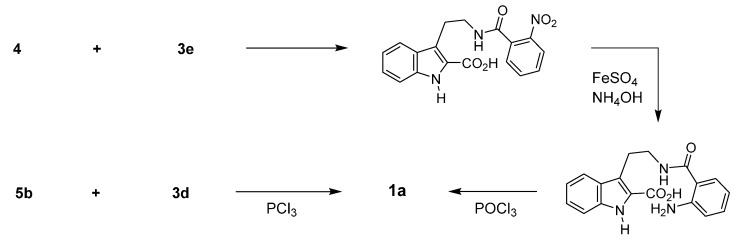
Synthesis of rutaecarpine by Asahina *et al*. [25,26].

On the other hand, a series of benzoic acid derivatives **3b**–**i** with nitrogen at the *ortho*-position (Figure 3) were employed as an equivalent for **3a** as the counterparts for tryptamine. In fact, in 1927 Asahina *et al*. reported two synthetic procedures for the synthesis of rutaecarpine using these equivalents as a starting materials—one procedure involved a three-step synthesis from 3-(2-aminoethyl)indole-2-carboxylic acid (**4**) and 2-nitrobenzoyl chloride (**3e**) (yield not given) [25] and the other a one-pot synthesis (in 24% yield) from 1,2,3,4-tetrahydro-1-oxo-β-carboline (**5b**) and methyl anthranilate (**3d**) in the presence of PCl_3_ [26] (Scheme 2).

Since the classification of syntheses in our previous review [1] was based on the structures of starting materials, we kept the same classification in the present review, that is: (1) tryptamine-derived syntheses; (2) tetrahydro-β-carboline-derived syntheses; and (3) miscellaneous.

### 2.1. Synthesis Using Tryptamine

**Scheme 3:**
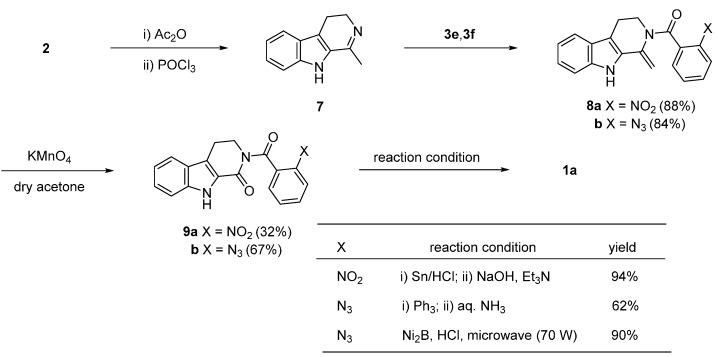
Synthesis of rutaecarpine by Lee *et al*. [27,28] and Kamal *et al*. [29].

Lee *et al*. [27,28] and Kamal *et al.* [29] used a one-pot reductive-cyclization of nitro (**9a**) and azide compounds (**9b**), respectively, to construct the quinazolinone skeleton. Tryptamine was subjected to a Bischler-Napieralsky reaction to afford starting compound **7**, which was then condensed with **3e** and 2-azidobenzoyl chloride (**3f**) to afford **8a** and **8b**, respectively. Cleavage of the exocyclic double bond led to the corresponding ketone **9**. It is worth noting that cleavage of the exocyclic double bond on **8** by ozonolysis failed, whereas oxidative cleavage with KMnO_4_ lead to ketones **9a** and **9b** in 32% and 67% yield, respectively (Scheme 3).

The reduction of the nitro group in **9a** by tin chloride resulted in subsequent cyclization giving **1a** in 94% yield [27,28]. On the other hand, the related 2-azobenzamide (**9b**) undergoes an aza-Wittig reductive cyclization in the presence of Ph_3_P and NH_4_OH or Ni_2_B in HCl-MeOH under microwave irradiation [29].

**Scheme 4:**
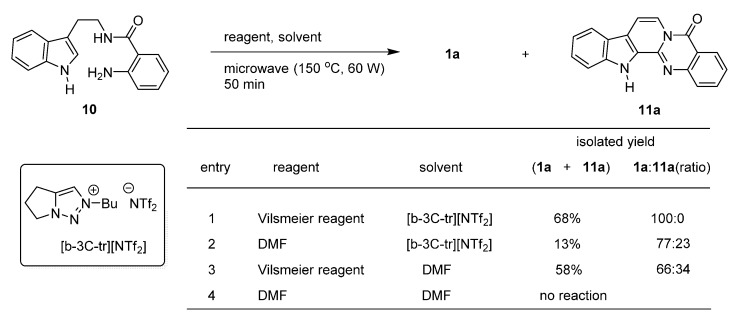
Synthesis of rutaecarpine by Tseng *et al*. [30].

Tseng *et al*. studied the potential use of bicyclic 1,2,3-triazolium ionic liquids for the synthesis of rutaecarpine from a one-carbon unit reagent and **10** [30]. Microwave-assisted cyclization of **10** led to **1a** and 7,8-dehydrorutaecarpine (**11a**) in ratios dependent on the reaction conditions (Scheme 4). The starting material **10** was prepared from tryptamine and isatoic anhydride (**3h**) in over 90% yield.

**Scheme 5:**
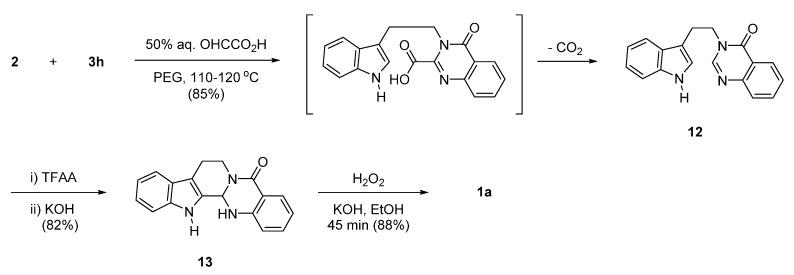
Synthesis of rutaecarpine by Rao *et al*. [31].

Rao *et al*. used 50% aqueous glyoxylic acid as a replacement for DMF or the Vilsmeier-Haack reagent in the above reaction. Reaction of **2** and isatoic anhydride (**3h**) in the presence of 50% aqueous glyoxylic acid led to **12**, which was then subjected to acid-catalyzed cyclization followed by H_2_O_2_/KOH-catalyzed dehydrogenation to produce rutaecarpine (**1a**) [31] (Scheme 5). Although the authors did not mention a possible reaction mechanism, the high reaction temperature would lead to decarboxylation of the possible intermediate 3-[2-(1*H*-indol-3-yl)ethyl]-4-oxo-3,4-dihydroquinazoline-2-carboxylic acid, to produce **12**.

**Scheme 6:**
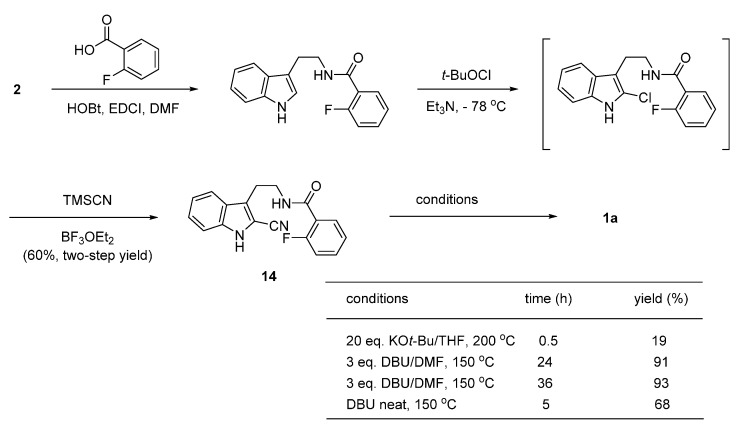
Synthesis of rutaecarpine by Liang *et al*. [33].

More recently, base-initiated intramolecular anionic cascade cyclization [32] of the 2-cyano compound **14** was applied to rutaecarpine synthesis by Liang, *et al*. [33]. The authors optimized the reaction conditions and found DBU was the reagent of choice for the conversion. The prerequisite 2-cyanoindole compound **14** was prepared in two-steps from tryptamine and 2-fluorobenzoic acid via a 2-chloroindolenine, generated by an electrophilic aromatic substitution reaction at the C_2_ position in the indole moiety by *t*-butyl hypochlorite, followed by nucleophilic substitution of the 2-chloro group by cyanide anion in the presence of BF_3_ (Scheme 6).

### 2.2. Synthesis Using Tetrahydro-β-Carboline

**Scheme 7:**
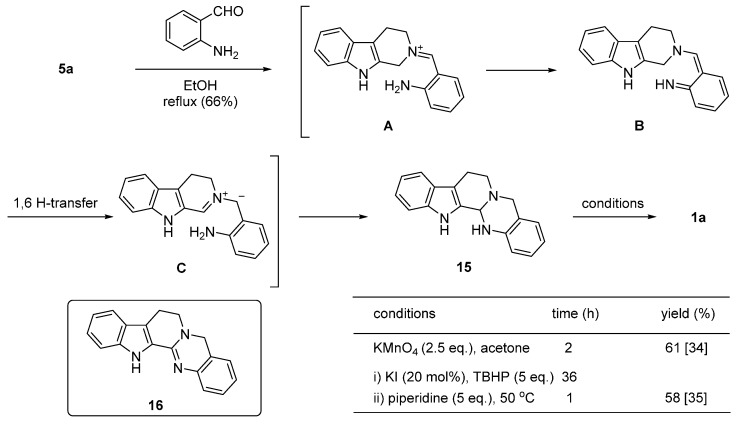
Synthesis of rutaecarpine via aminal [34,35,36].

Zheng *et al*. reported the oxidation of s ring-fused aminal to rutaecarpine via an α-amination of an *N*-heterocycle as the key reaction [34,35,36] (Scheme 7). The α-position of 1,2,3,4-tetrahydro-β-carboline (**5a**) was activated by reacting with 2-aminobenzaldehyde to form an iminium ion (**A**), which was converted to the quinonoidal intermediate (**B**) by rearrangement of adjacent π-systems and a proton loss. 1,6-Hydrogen atom transfer in **B** led to the dipolar intermediate **C**, which ultimately afforded the cyclized aminal product (**15**). Oxidation of **15** by KMnO_4_ afforded rutaecarpine (**1a**) in 61% yield (Scheme 7). It should be noted that the MnO_2_ oxidation of dehydroaminal such as **16** yielded **1a** and fully dehydrogenated product (**11a**) in an 8:1 ratio [37].

**Scheme 8:**
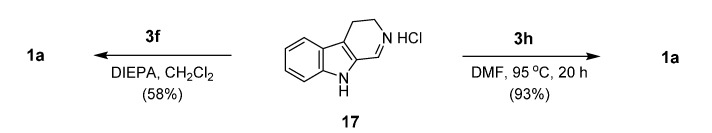
Synthesis of rutaecarpine using 3,4-dihydro-β-carboline [38,39].

On the other hand, reaction between 3,4-dihydro-β-carboline (**17**) and *o*-azidobenzoyl chloride (**3f**) in the presence of Hünig’s base delivered **1a** in 58% yield [38], while reaction with **3h** afforded **1a** in 93% yield [39] (Scheme 8).

### 2.3. Miscellaneous

**Scheme 9:**
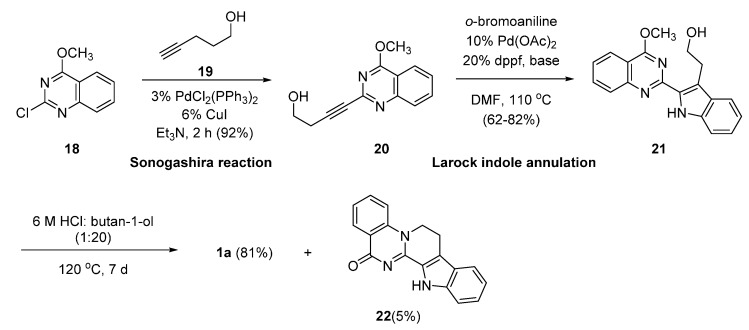
Synthesis of rutaecarpine by Pan and Bannister [41]

Synthetic methods not employing anthranilic acid, tryptamine, or their equivalents are very rare. Recently, Pan and Bannister employed a sequential Sonogashira reaction and Larock indole synthesis, whereby Sonogashira’s Pd(0)-catalyzed ethynylation of **19** to **18** led to **20**, which subsequently underwent an intramolecular Pd(0)-catalyzed indole formation [40] to produce **21**. The acid-catalyzed cyclization of **21** led to **1a** in 81% yield with a trace of isomeric compound **22** [41] (Scheme 9). To the best of our knowledge, this procedure is the first example of construction of the C-ring to synthesize rutaecarpine via N_6_-C_7_ bond formation.

To synthesize rutaecarpine, C-ring construction can be pursued in three different ways via: (1) C_13a_-C_13b_ bond formation; (2) N_6_-C_7_ bond formation; or (3) C_8_-C_8a_ bond formation. Methods involving C_13a_-C_13b_ bond formation at the final stage of synthetic sequences have been most commonly used [1,26,30,31,42,43,44,45,46,47]. However, a method employing C_8_-C_8a_ bond formation has not been studied as yet.

### 2.4. Synthesis of Bioisosteres and Hybrid Rutaecarpine Systems

**Scheme 10:**
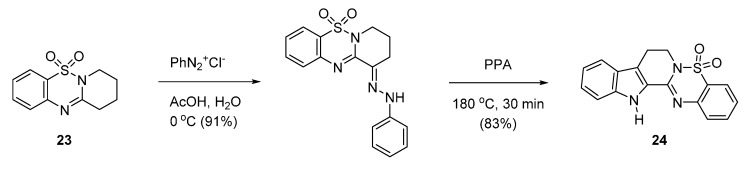
Synthesis of 5-sulfarutaecarpine [48].

Bioisosteric replacement of the quinazolinone moiety of the pentacyclic system with benzothiadiazine 1,1-dioxide has been pursued [48] (Scheme 10). Bubenyák *et al.* reported a condensation of benzothiadiazine 1,1-dioxide analogue **23** with phenyldiazonium chloride led to a phenylhydrazone derivative, which was subjected to Fischer indole synthesis to afford the corresponding 5-sulfarutaecarpine (**24**). The starting **23** was prepared in two-steps from 2-aminobenzenesulfonic acid and 6-methoxy-2,3,4,5-tetrahydropyridine in 33% yield or 2-nitrobenzenesulfonyl chloride and piperidin-2-one in 66% yield.

**Scheme 11:**
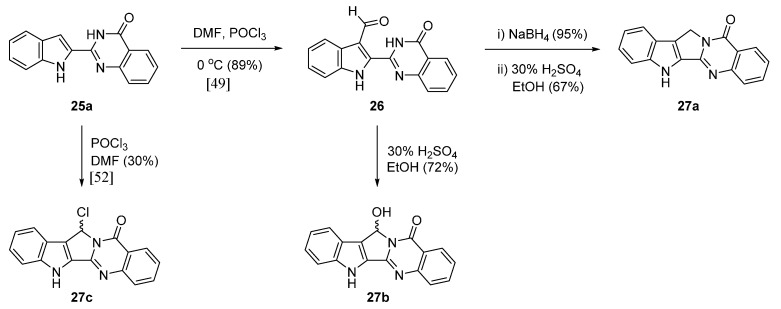
Synthesis of a hybrid between rutaecarpine and luotonin A [50,52].

The same group [49] reported a hybrid between the alkaloids rutaecarpine and luotonin A [50,51]. Vilsmeier-Haack formylation of 2-(1*H*-indol-2-yl)quinazolin-4(3*H*)-one (**25a**) gave **26** which was subjected to either direct or indirect reduction to alcohol followed by acid catalyzed cyclization to produce **27a**,**b**. On the other hand, a direct cyclization followed by chlorination under Vilsmeier-Haack conditions led to **27c** [52] (Scheme 11). Such a chloro-compound represents a good substrate for introducing substituents by nucleophilic substitution [52] for the synthesis of related series of compounds.

## 3. Biological Properties

A review written in 2003 by Hu and Li, comprehensively described the *in vitro* and *in vivo* pharmacology of rutaecarpine [18], in which pharmacological actions were classified as; cardiovascular effects, antiplatelet activity, antithrombotic activity, anticancer activity, anti-inflammatory and analgesic effects, effects on the endocrine system, anti-obesity and thermoregulatory effects, effects on smooth muscle (except cardiovascular), and others. In addition, rutaecarpine ameliorated body weight gain by inhibiting orexigenic neuropeptides NPY and AgRP in mice [53] and reducing lipid accumulation by AMPK (AMP activated protein kinase) activation and UPR (unfolded protein response) suppression [52]. Recently, Xu *et al*. reported the anti-atherosclerosis activity (EC_50_ = 0.27 μM) by up-regulating ATP-binding cassette transporter A1 (ABCA1) [54,55]. The present review addresses structure-activity relationships with respect to antiplatelet activity, vasodilator activity, cytotoxicity, and anticholinesterase activity.

### 3.1. Antiplatelet Activity

Early studies revealed that the antiplatelet activity of rutaecarpine was due to the inhibition of thromboxane formation and phosphoinositide breakdown [56]. Two different antiplatelet activities (85.2% aggregation at the concentration of 100 μg∙mL^−1^ [28] *vs.* 0% aggregation at the concentration of 20 μg∙mL^−1^ [57]) have been reported for rutaecarpine, whereas 2,3-methylenedioxyrutaecarpine (**1b**), 3-chlororutaecarpine (**1c**), and 3-hydroxyrutaecarpine (**1d**, IC_50_ = 1–2 μg∙mL^−1^) showed 100% inhibition towards arachidonic acid-induced aggregation at 5 μg∙mL^−1^. However, aggregations induced by ADP (0.22 μM), thrombin (0.1 unit∙mL^−1^), collagen (10 μM), and platelet activating factor (PAF, 2 μg∙mL^−1^, data not shown) were not affected by rutaecarpine or its derivatives except 3-methoxyrutaecarpine (**1e**, 19.8% aggregation at 100 μg∙mL^−1^ level) and butanoic acid derivative (**1a**, **b**, 19.8% aggregation at 100 μg∙mL^−1^). Results are summarized in Table 2.

**Table 2:** Inhibitory activity of rutaecarpine and its derivatives on the platelet aggregation.
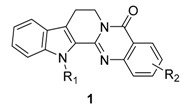

Compound	R_1_	R_2_	Aggregation (%)			
			Inducer			
			ADP (0.22 μM)	Thrombin (0.1 unit∙mL^−1^)	A.A. ^a^ (100 μM)	Collagen (10 μg∙mL^−1^)
**1a**	H	H	99 ^b^ [56]	85.7 ^c^ [28]	85.2 ^c^ [28], 0 ^d^ [57]	82.1 ^c^ [28], 22.1 ^c^ [57]
**1b** [28]	H	2,3-OCH_2_O-		89.8 ^b^	0 ^e^	
**1c** [28]	H	3-Cl		88.8 ^b^	0 ^e^	75.4 ^c^
0.5% DMSO [28]				88.2	90.1	88.5
**1d** [58]	H	3-OH		84.0 ^d^	0 ^e^	51.1 ^c^
**1e** [58]	H	3-OMe		89.7 ^e^	0 ^f^	19.8 ^c^
aspirin (20 μg∙mL^−1^) [58]				92.1 ^d^	0 ^e^	90.1 ^d^
**1aa** [57]	CH_2_CH_2_OH	H	76 ^b^			
**1ab** [57]	(CH_2_)_3_CO_2_H	H	14 ^b^			
**1ac ** [57]	3,4-(Me)_2_C_6_H_3_	H	88 ^b^			

The values were given with standard error of the mean (SEM), but intentionally omitted SEM for clarity. Platelets were pre-incubated with 0.5% DMSO (control) or compounds for 3 min, then the inducer was added to trigger aggregation. ^a^ A.A. = arachidonic acid; ^b,d,e,f^ Values given were aggregation percentage in the presence of 100, 20, 5, and 50 μg∙mL^−1^ of rutaecarpines, respectively; ^c^ Aggregation percentage at the concentration of 10 μM.

### 3.2. Vasodilator Activity

An early study showed that phenylephrine-induced contraction of isolated rat mesenteric arterial segments with intact endothelium was relaxed by 90% by 0.1 mM rutaecarpine and that such relaxation was concentration-dependent in the 0.1 μM–0.1 mM range [59]. Further study revealed that NO-dependent vasodilation is primarily responsible for the vasodilatory activity of rutaecarpine [60]. Subsequently, the vasodilatory effect of rutaecarpine was also related to the stimulation of endogeneous calcitonin gene-related peptide (CGRP) release via the activation of transient receptor potential vanilloid subfamily, member 1 (TRPV1) [61,62]. Chen *et al*. synthesized 12 rutaecarpine derivatives and 11 analogues, and then evaluated their vasodilator activities (data not shown). These authors found two important trends regarding the vasodilator activities of rutaecarpine-related anti-hypertensives: (1) the N_14_ atom of rutaecarpine might be the key site, and (2) the 5-carbonyl probably makes a lower contribution, while simple substitution on the indole or quinazoline rings does not enhance vasodilatory effects [62]. Although the prepared compounds showed better activity than rutaecarpine (EC_50_ = 1.33 μM), such a finding would suggest a new direction for the discovery of valuable TRPV1 agonists as anti-hypertensive drugs if rutaecarpine had proper substituent (s) at the proper position (s).

### 3.3. Cytotoxicity

#### 3.3.1. Rutaecarpine Derivatives

The findings from the studies on the cytotoxicity of rutaecarpine and its derivatives are summarized in Table 3. In general, ring substitution results in selectivity towards specific cell lines. Although the introduction of substitutions on ring A affected the cytotoxicity more significantly, the position is also important. 11-Methoxyrutaecarpine (**1f**) showed selective cytotoxicity against a human lung and renal cancer cells at concentrations of 0.75 and 0.31 μM, respectively, and the 10,11-methylenedioxy analogue **1b** showed selective cytotoxicity for ovarian cancer cell lines, while 10-methoxyrutaecarpine (**1e**) showed no significant cytotoxicity at concentrations up to 25 μM [63]. 12-Fluororutaecarpine (**1n**) showed somewhat stronger cytotoxicity in the HT-29 human cell line compared to 2-chlororutaecarpine (**1m**) and those with fluorine on ring E (data not shown) [64]. 11,12-Dichlororutaecarpine (**1o**), a hybrid of bauerine (7,8-dichloro-9-methyl-2,9-dihydro-1*H*-pyrido[3,4-*b*]indol-1-one) [65] and rutaecarpine, showed the strongest inhibitory activity against HL-60 at the 0.15 μM level.

Regarding the mechanisms responsible for cytotoxicity, inhibitory activities against topoisomerase (topo) I and II have been studied. These inhibitory activities appeared to be affected by substitution on the E-ring but not by substitutions on rings A and/or C [64,66,67], except for 11-bromorutaecarpine. In fact, 10-bromorutaecarpine (**1g**) and 3-chlororutaecarpine (**1c**) showed strong inhibitory activities (79.54% and 84.35%, respectively) against topo I and were comparable to camptothecin (82.62%) at 100 μM against 0.2 unit topo I and similar to that against 0.2 units of topo II [67] (see Table 5). In addition, rutaecarpine inhibited tumor cell migration by approximately 30%–40% at 100 μg∙mL^−1^ [68], which would open a new study-window on the use of rutaecarpine as an antitumor agent.

#### 3.3.2. Rutaecarpine-Isosteres and Hybrids

Annulation of aromatic rings, especially thiophene (**27a**–**d**), pyrrole (**27e**), and furan (**27f**) rings onto rutaecarpine ring E enhanced cytotoxicity toward selected human cancer cell lines [69]. All of these systems showed improved cytotoxicity against melanoma (UACC62), ovarian (SKVO3), prostate (DU145) and renal cancer (ACHN) cell lines but not against CNS or lung cancer cell lines.

On the other hand, dihydrorutaecarpines (**29**) showed increased activity and selectivity toward a CNS (U251) cancer cell line in the concentration range 0.02–7 μM and toward a renal cancer cell line at 0.08–20 μM (Table 4). The parent **30a** showed strong activity (GI_50_ = 0.02 μM) and selectivity toward a CNS cancer cell line and the 10-methylthio compound (**29b**) showed strong activity (GI_50_ = 0.08 μM) and selectivity toward a renal (ACHN) cancer cell line.

It is worth noting that evodiamine (**29h**) showed strong *in vitro* cytotoxicity against the following cell lines; human leukemia (HL-60, GI_50_ = 0.51 μM), human prostate cancer cell line (PC-3, GI_50_ = 14.35 μM) [70], murine fibrosarcoma (L929, GI_50_ = 20.3 μM), human breast adenocarcinoma (HeLa, GI_50_ = 15.4 μM), and human malignant melanoma (A375-S2, GI_50_ = 10.1 μM) [71].

**Table 3:** *In vitro* cytotoxicity of rutaecarpine derivatives (GI_50_ values in μM) ^a^.
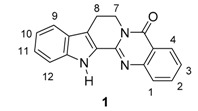

	SF-295	HT-29	A549/ATCC	NCI-H460	OVCAR-4	786-0	CCRF-CEM/HL-60	N87	HS-578T
**1a** (rutaecarpine)	14.1 [72]	31.6 [73]	14.5 [63]		18.9 [63]		19.8 [74]	8.41 [74]	22.6 [63]
**1b** (10,11-OCH_2_O-) [63]			>25.0	1.55	1.50	1.08	>25.0		5.05
**1d** (3-OH) [70]							11.94		
**1e** (10-OCH_3_) [63]			>25.0	>25.0	>25.0	>25.0			>25.0
**1f** (11-OCH_3_) [63]			0.75	1.38	>25.0	0.31			1.59
**1g** (10-Br) [72]	8.62		16.3 ^b^		6.43(11.1) ^c^				
**1h** (1-OH) [73]		7.39	10.43				10.1 [74], 8.34 [70]	8.38 [74]	
**1i** [7-OH(β)] [74]							10.1	23.2	
**1j** [7-OH(β), 8-OH(α)] [74]							13.7	14.1	
**1k** [7-OH(β), 8-OCH_3_(α) [74]							7.82	22.3	
**1l** [7-OH(β), 8-OEt(α)] [74]							8.31	27.9	
**1m** (2-Cl) [64]		5.62	22.4						21.6 ^d^
**1n** (12-F) [64]		1.26	8.4						3.18 ^d^
**1o** (11,12-diCl) [75]							0.15		

Tumor cell lines: CNS cancer (U251), human colon carcinoma (HT-29), human lung adenocarcinoma (A549/ATCC and NCI-11460), renal cancer (786-0, ACHN), ovarian cancer (OVCAR-4), leukemia (CCRF-CEM, HL-60, or P-388), human gastric carcinoma (N87), and breast cancer (HS-578T). ^a^ The cytotoxicity GI_50_ values are the concentrations corresponding to 50% growth inhibition, and they are the averages of at least two determinations; ^b^ for non-small cell lung cancer cell line; ^c^ Value in parenthesis was taken from ovarian OVCAR-8 cancer cell line; ^d^ for human breast cancer carcinoma MCF-7.

**Table 4:** *In vitro* cytotoxicity activities of fused-rutaecarpines and dihydrorutaecarpines (GI_50_ values in μM) ^a^ [69].
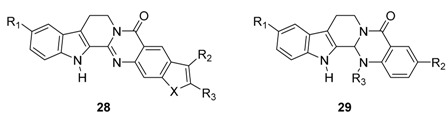

Compound	X	R_1_	R_2_	R_3_	U251	H522	UACC62	SKOV3	DU145	ACHN
**28a**	S	H	H	H	>100	>100	7	10	>100	>100
**28b**	S	Cl	H	H	53	74	2	12	12	2
**28c**	S	SCH_3_	CH_3_	CO_2_Et	>100	>100	7	20	2	13
**28d**	S	F	H	H	>100	>100	7	17	1	46
**28e**	N	Br	CH_3_	H	26	33	9	14	34	20
**28f**	O	Cl	*t*-Bu	H	11	-	14	8	15	2.4
**29a**	-	H	H	H	0.02	37	>100	3	0.2	20
**29b**	-	SCH_3_	H	H	3	35	17	13	15	0.08
**29c**	-	SOCH_3_	H	H	7	>100	>100	27	0.1	2
**29d**	-	Br	H	H	5	59	>100	>100	32	1
**29e**	-	Cl	H	H	2.5	9	>100	2	2	2
**29f**	-	Cl	H	CH_3_	6	10	7	11	2	1
**29g**	-	Br	H	CH_3_	0.3	18	>100	3	3	0.4
**29h**	-	H	H	CH_3_	5	1	6	2	15	11
**29i**	-	H	NO_2_	H	3	3	2	12	3	3

Most of the values were given with standard error of the mean (SEM), but intentionally omitted SEM for clarity. Tumor cell lines: CNS cancer (U251), lung cancer (H522), melanoma cancer (UACC62), ovarian cancer (SKOV3), prostate cancer (DU145), and renal cancer (ACHN). ^a^ The cytotoxicity GI_50_ values are the concentrations corresponding to 50% growth inhibition, and are the averages of at least two determinations.

Although the cytotoxicity of 13*b*,14-dihydrorutaecarpine (**29a**) is somewhat more potent than that of rutaecarpine (**1a**), it is not easy to establish any possible structure-activity relationship between 13*b*,14-dihydrorutaecarpine derivatives **29** and the corresponding rutaecarpine derivatives **1**, not only because the substitution patterns are different but also because tested cancer cell lines are different.

The poor solubility of rutaecarpine and its derivatives in common organic solvents and in water results in poor bioavailability, which is the main obstacle that needs to be resolved for the further development of rutaecarpine and its derivatives as a drug. In fact, the excellent *in vitro* activities (GI_50_ = 1–8 μM) of compounds **29e**, **29f**, and **29i** were not reflected by xenograft model results, presumably because of their poor bioavailability [69].

Generally, benzo-annulation increases electronic dispersion and planar dimensions thus may play an important role in interactions with receptor sites [76,77]. A series of benzo-annulated rutaecarpines were prepared using Fischer indole synthesis and their cytotoxicities against selected human cancer cell lines and their inhibitory effects on topo I and II were evaluated [78] (Table 5). However, currently available data are not sufficient to indicate any clear structure-activity relationships.

It should be noted that an isostere **24** with a sulfone moiety was not as active as rutaecarpine. The percentage of apoptotic cells corresponding to the sub-GI phase of 5-sulfarutaecarpine was found to be 29.2%, which compares with the 14.5% of rutaecarpine [48]. In addition, the hybrids **27a** showed an increase of cytotoxic activities against HeLa cells and apoptosis inducing effects at a concentration comparable to that of etoposide. The percentages of apoptotic cells corresponding to the sub-G1 phase of **26** and **27a** at the 10^−6^ mol∙L^−1^ were 38.6% and 24.1%, respectively, which are comparable to the 14.5% of rutaecarpine while a positive control (etoposide) gave 15.4%.

**Table 5:** *In vitro* cytotoxicity activities of benzorutaecarpines (GI_50_ values in μM) ^a^ [78].
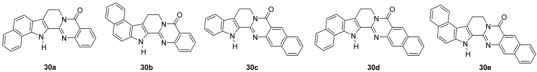

Compound	Cell Lines (GI_50_, μM)					Topo I Inhibition (%)	Topo II Inhibition (%)
	HEK293	MCF7	DU145	HCT116	K562		
**1a**	>50	19.57	31.65	33.89	25.77	1.20	1.25
**1g**	(see Table 3)				79.54	35.43
**1c**	NT	NT	NT	NT	NT	84.36	69.51
**30a**	36.57	11.42	27.54	9.94	25.38	NA	45.38
**30b**	>50	16.94	36.55	24.16	31.42	48.09	3.52
**30c**	>50	12.25	30.14	15.78	25.63	0.46	45.92
**30d ** ^b^	-	-	-	-	-	-	-
**30e**	>50	12.06	29.64	25.36	28.39	28.11	52.74
etoposide ^c^	4.09	3.02	3.75	1.88	2.23	52.52	-
camptothecin ^c^	5.16	4.22	3.22	0.38	1.05	-	63.41

^a^ The original data were from three different experiments performed in triplicate and given as mean +/− standard error of the mean (SEM), but intentionally omitted SEM for clarity. Cell lines used are embryonic kidney cell line (HEK293), human breast cancer (MCF7), human prostate tumor (DU145), human colorectal adenocarcinoma (HCT116), and human chronic myelogenous leukemia cell line (K562); ^b^ Not soluble enough to produce meaningful values; ^c^ Data was taken with 0.2 unit of topo I (or II), 100 μM of reference (camptothecin or etoposide) and benzorutaecarpines prepared.

### 3.4. Inhibitory Activity on Acetylcholinesterase

The early studies on the strong inhibitory activity (64% inhibition at 100 μg∙mL^−1^) of fruits extract of *Evodia officinalis* against acetylcholinesterase [79] and its strong *in vivo* anti-amnesic activity (IC_50_ = 6.3 μM) led to the finding that dehydroevodiamine (**31**) was the origin of such biological activities [80] (Table 6 and Table 7). These results led to more systematic studies on the anti-cholinesterase activity of rutaecarpine [81].

Wang, *et al*. prepared a series of rutaecarpine (compounds **1q**–**w**) (Table 6) and 7,8-dehydrorutaecarpine (**11**, *vide infra*) derivatives (Table 7). Most of the rutaecarpine derivatives showed strong inhibitory activity against acetylcholinesterase (*ee*AChE) from electric eel and butylcholinesterase (*eq*BuChE) from equine serum with a selectivity on AChE over BuChE in the range 0.1–297.5 [81]. Additional structural modifications of **11** lead a dramatic increase in anticholinesterase activity up to 0.61 nM (**11h**) and selectivity on AChE up to over 3000 [81,82].

**Table 6:** *In vitro* inhibition and selectivity index of rutaecarpine derivatives on *ee*AChE and *eq*BuChE.
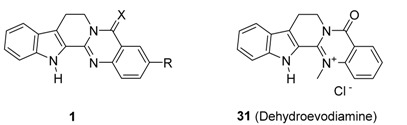

Compound	R	X	AChE Inhibition (nM) ^a^	BuChE Inhibition (nM) ^b^	Selectivity Index ^c^
**1a**	H	O	>100,000	>100,000	
**1q**	NHCOCH_2_NEt_2_	O	372.3	17,620	47.4
**1r**	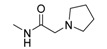	O	131.2	696.1	5.3
**1s**	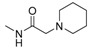	O	111.4	33,020	297.5
**1t**	NHCO(CH_2_)_2_NEt_2_	O	80.20	2848	35.5
**1u**	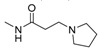	O	29.24	844.5	28.9
**1v**	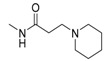	O	21.40	2112	98.7
**1w**	H	H,H	340	50	0.15
tacrine			222.7	29.98	0.1
**31 **[79]			13,200 (37,800 [80], 6300 [83])	115,900	8.8

The original data were given as mean +/− standard error of the mean (SEM), but intentionally omitted SEM for clarity. ^a^ 50% inhibitory concentration (means of at least four independent experiments) of *ee*AChE from *electric eel*; ^b^ 50% inhibitory concentration (means of at least four independent experiments) of *eq*BuChE from *equine serum*; ^c^ Selectivity Index for AChE = IC_50_ (BuChE)/IC_50_ (AChE).

**Figure 4:**
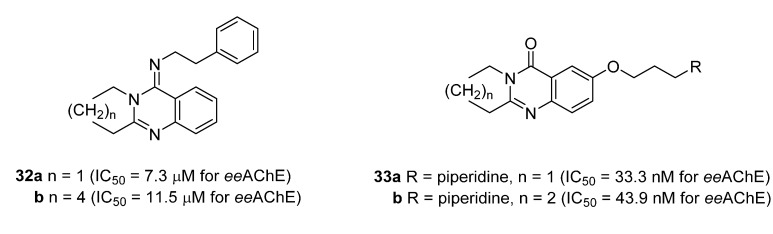
Selected examples of truncated rutaecarpines.

Interestingly, **5b**, not only natural product but also one of the favored starting materials for rutaecarpine synthesis, showed promising inhibitory activity on AChE (IC_50_ = 83.38 μM) [84], implying a new vista towards designing new analogues of rutaecarpine for the treatment of Alzheimer’s disease. In addition to **5b**, studies on truncated rutaecarpines have led to new promising lead compounds, such as **25** [85] (Table 8), **32** [86], and **33** [87] (Figure 4).

**Table 7:** *In vitro* inhibition and selectivity index of dehydrorutaecarpine derivatives on *ee*AChE and *eq*BuChE [81,82].
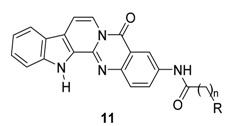

Compound	R	N	AChE Inhibition (nM) ^a^	BuChE Inhibition (nM) ^b^	Selectivity Index ^c^
**11b** [81]	NHCOCH_2_NEt_2_		57.09 (70.4 [82])	11,360	198.9
**11c** [81]	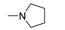	1	23.56 (59.3 [82])	428.2	18.2
**11d** [81]	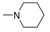	1	10.07 (70.0 [82])	5429	539.1
tacrine [81]		222.7	29.98	0.1	
**11e** [82]	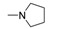	2	13.90	14,900	1072
**11f** [82]	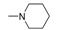	2	51.00	24,000	471
**11g **[82]	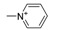	1	0.80	2451	3225
**11h** [82]	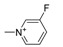	1	0.61	1855	3092
**11i** [82]	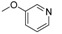	1	3.09	7300	2362
**11j **[82]	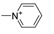	2	2.30	4291	1858
**11k** [82]	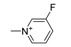	2	2.10	3488	1638
**11l** [82]	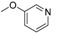	2	14.30	6340	450
**11m** [82]	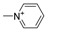	3	3.90	4160	1056
tacrine [82]		108.0	33.4	0.3	

The original data were given as mean +/− standard error of the mean (SEM), but intentionally omitted SEM for clarity. ^a^ 50% inhibitory concentration (means of at least four independent experiments) of *ee*AChE from *electric eel*; ^b^ 50% inhibitory concentration (means of at least four independent experiments) of *eq*BuChE from *equine serum*; ^c^ Selectivity Index for AChE = IC_50_ (BuChE)/IC_50_ (AChE).

The anticholinesterase activity and the selectivity on AChE were somewhat related to the back-bone structure and the length of the side chain: The derivatives with a backbone with an aromatic C ring (**11**) showed better activity and selectivity than non-aromatic (**1**) and open-chain systems (**25**). The 7,8-dehydrogenated rutaecarpine derivative (**11c**, IC_50_ = 23.56 nM, SI = 18.20) is more active and more selective than the rutaecarpine derivative (**1r**, IC_50_ = 131.2 nM, SI = 5.3) and open C-ring derivative (**25b**, 326.6 nM, SI = 9.50) (Table 8). On the other hand, an increase of the length of the side chain would increase the activity (**1q** *vs.* **1t**, **1r** *vs.* **1u**, and **1s** *vs.* **1v**; **11c** *vs.* **11e**; and **25c** *vs.* **25d**) and selectivity except in the case of **1s** *vs.* **1v**.

The selectivity indexes (SI, calculated by IC_50_ for BuChE/IC_50_ for AChE) on AChE of all the rutaecarpine derivatives ranged from 5.3–3225. Although the selectivity on AChE over BuChE is a concern for curing Alzheimer’s disease, clinically useful physostigmine (SI = 3.47) [84], galanthamine HBr (SI = 13.1 [86]) and donepezil (SI = 1252 [88]) show selectivity for AChE over BuChE while rivastigmine (SI ≤ 0.008 [86]) and neostigmine (SI = 0.58 [89]) show selectivity for BuChE over AChE. These results imply that it may not be an advantage for a cholinesterase inhibitor to be selective for AChE or BuChE, but instead suggest that higher efficacy requires a good balance between AChE and BuChE.

**Table 8:** *In vitro* inhibition and selectivity index of truncated-rutaecarpines on AChE and BuChE [84,85].
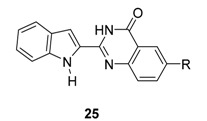

Compound	R	AChE Inhibition (nM) ^a^	BuChE Inhibition (nM) ^b^	Selectivity Index ^c^
**5b** [84]		83,380		
physostigmine [84]		170	590	3.47
**25a** [85]	H	>10,000	>10,000	
**25b** [85]	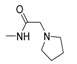	326.6	3103	9.50
**25c** [85]	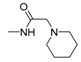	147.9	10,160	68.70
**25d** [85]	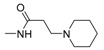	20.98	7322	349.17
tacrine [85]		222.7	29.98	0.1

The original data were given as mean +/− standard error of the mean (SEM), but intentionally omitted SEM for clarity. ^a^ 50% inhibitory concentration (means of at least 3 independent experiments) of *ee*AChE from *electric eel*; ^b^ 50% inhibitory concentration (means of at least 3 independent experiments) of *eq*BuChE from *equine serum*; ^c^ Selectivity Index for AChE = IC_50_ (BuChE)/IC_50_ (AChE).

## 4. Conclusions

Rutaecarpine is one of the important alkaloids isolated from the Rutaceae and related plants, and it exhibits various interesting biological properties. Recent years have witnessed steady progress in understanding the chemistry and biology of rutaecarpine. Furthermore, it should be noted that reports have been issued on the beneficial effects of rutaecarpine analogues on controlling lipid accumulation [52], obesity [53], and atherosclerosis [54,55], and. The present review focuses on the synthesis of rutaecarpine derivatives and on their biological activities, especially on structure-activity relationships and their antiplatelet, vasodilatory, cytotoxic, and anticholinesterase activities. More efficient and/or practical methods are needed for the synthesis of rutaecarpine derivatives, not only to pursue structure-activity relationship studies but also to identify novel potent lead compounds for drug development.
